# Can digital farming technologies enhance the willingness to buy products from current farming systems?

**DOI:** 10.1371/journal.pone.0277731

**Published:** 2022-11-14

**Authors:** Rolf Wilmes, Gabi Waldhof, Peter Breunig

**Affiliations:** 1 Weihenstephan-Triesdorf University of Applied Sciences, Triesdorf, Germany; 2 Department of Structural Change, Leibniz Institute of Agricultural Development in Transition Economies, Halle, Germany; Canakkale Onsekiz Mart University, TURKEY

## Abstract

While current global agriculture allows for efficient food production, it brings environmental disadvantages, which resulted in a lack of public acceptance. Digital technologies (e.g., technologies that enable precision agriculture) have been suggested as a potential solution to reconcile environmental sustainability and yield increases. By embedding digital technologies into holistic farming system visualized through mission statements, this study tests the effect of different intensities of digitization, as well as environmental arguments on the willingness to buy food produced by farms in Germany. We use a 4 x 4 repeated measure experimental design surveying a representative sample of 2,020 German citizens recruited online. Our research framework captures the farming system (comparing low intensity of digitalization for a small or organic farm and low, medium, and high intensity of digitalization for large or conventional farms) and environmental arguments (comparing no arguments, and altruistic, egoistic, and biospheric arguments). The results show a negative effect of digital technologies on willingness to buy. However, this relationship turns positive when introducing environmental arguments. Furthermore, there is a moderation effect for respondents’ attitudes towards technologies that varies depending on whether altruistic, egoistic, or biospheric concerns were stated. The results indicate that digital technologies can increase willingness to buy products from both large and conventional farms, but not to the level of small farms and organic farms.

## Introduction

Modern agriculture is faced with the challenge of feeding a growing world population while at the same time ensuring environmental sustainability. Through intensification and industrialization of agriculture, which came along with the consolidation of farms and intensive use of synthetic agrochemicals, food production could be enhanced [1]. Still prospectively, the agricultural sector needs to further intensify to keep up with increasing demand [2, 3]. However, unfortunately, this increase in food production came at the price of negative environmental impacts [4] which resulted in lower public acceptance of these practices [5].

Contrary to that, small-scale farming and organic agriculture are alternatives to intensification and industrialization that consider consumer´s preferences such “natural” production [6, 7]. Consequently, these practices enjoy higher public acceptance. However, recent research has questioned whether small-scale operations [8] and organic farming [9] enable sustainable intensification (SI), i.e., increasing food production from existing farmland while at the same time reducing environmental impacts [10]. Newly introduced methods of digital agriculture (DA) may offer a solution that reconciles both, environmental friendliness and increased yield levels.

Because DA provides a means to SI [10–12], we here address the question of whether these methods of DA also increase the public acceptance of modern agriculture.

Public acceptance, understood here as the degree to which a public (e.g., of a specific country) is willing to purchase products made through certain practices or technologies, plays a crucial role in policy-making and technology adoption [13]. Despite the important role of DA in striving towards SI, research on public acceptance related to DA is limited. Recently, scholars have started to examine public perception of digital technologies [14] and, recently, public acceptance of single digital technologies [15]. To date, the literature has not clearly delimited what drives the public acceptance of DA. The problem statement of this research is to answer the question whether DA can increase public acceptance of intensive farming systems up to levels similar to those of organic farms and small family farms. As mentioned above these two farm types enjoy high public acceptance and are therefore chosen as a reference to measure the impact of DA on public acceptance.

To examine the impact of DA on the acceptance of agriculture relative to farm size (small family farm vs. large professional farm) and production method (organic production vs. conventional production), this paper builds on research on the acceptance of novel food technologies. This stream of research has broadened the understanding of the importance of technical features and their respective relevance for individual perception and thus acceptance [13, 16], measured through assessing the willingness to buy (WTB) products produced by certain technologies [17, 18]. As such, general attitudes towards technologies have been found as important determinants of individual perception, shaping how people justify new technologies. Furthermore, given the interplay between agriculture and its environment, the evaluation of technical features regarding DA might be influenced by environmental concerns, which [19] conceptualized as altruistic, egoistic, and biospheric value orientations. For example, people evaluate a new technology according to the extent to which it “tampers with nature” [20]. Therefore, the purpose of this paper is to examine the impact of DA on the acceptance of agriculture relative to farm size and production method. In line with previous research, we use WTB products from a certain agricultural system as an indicator of public acceptance. Specifically, the extent to which the model of environmental concerns [19] holds for the acceptance of DA in agriculture is examined. This is accomplished through developing a research design that simplifies the understanding of complex DA by embedding digital technologies into holistic farming systems and utilizing mission statements for visualization. Each mission statement describes a farm by its size or production method and the degree to which technologies are utilized to grow crops on that farm. We distinguish between different intensities with which digital technologies are integrated into farming systems by describing the impact of technologies on 1) decision making, 2) automation, and 3) precision in arable farming. Embedding mission statements into a repeated measure design allow the trajectory of acceptance to be captured for different intensities with which technologies might find their way into arable agriculture.

This paper has several contributions to academic and farm managerial discussions. First, this paper contributes to knowledge on the public acceptance of modern agriculture by investigating the WTB of holistic farming systems that incorporate digital technologies. Through the assessment of varying degrees of digital technologies, one being a future scenario, effects on the acceptance of intensive agriculture can be estimated. This is particularly relevant given that the adoption of digital technologies will likely continue to increase [21]. Second, the repeated measure research design enables the assessment of DA for public acceptance relative to two farm characteristics that are in the public discussion: farm size (small family farm vs. large professional farm) and production method (organic vs. conventional). Third, this paper contributes to research on the public acceptance of DA by introducing environmental concerns [19]. Considering altruistic, egoistic, and biospheric value orientations might help to better understand the circumstances under which communication strategies foster public acceptance of DA as their effectiveness can vary between consumers’ general attitudes towards technology [15]. Finally, this study has implications for policymakers and actors in the agricultural industry related to how public acceptance of future forms of agriculture can be strengthened. Policymakers should emphasize outcome-based judgment of food production to foster the acceptance of technologies beneficial for SI.

### Public acceptance of technologies and agriculture

Attitudes towards technologies in agriculture do not only depend on technical features but also on numerous individual perceptions, such as risk and benefit perceptions [15]. [16] proposed a framework in which attitudes towards technology are formed by its technical features that are evaluated through individuals’ perceptions (i.e., perceived risks or benefits). Technical features might have a low explanatory power since they are mediated through an individual’s underlying perceptions. A recent study by the [22] suggests that technological progress is widely perceived as positive by society. Consequently, recent advances and technological progress in DA might positively impact people’s WTB products from more digitalized farming systems (i.e., farming systems where the use of technology improves decision making, automation, and precision in arable farming) [14]. Therefore, the following hypothesis (H1a) is proposed: The extent of digital technologies in farming systems is positively associated with the WTB products from these systems.

In many European countries, the consolidation of farms has been a continuous process that began in the last century. Accordingly, the total number of farms has been declining while the remaining farms have, on average, become larger. While growing farm size can foster profitability [8], recent research shows external effects on the environment. For example, larger farms are associated with lower landscape diversity, thus mitigating biodiversity [23–25]. Consequently, food production by small family farms might be perceived as more natural compared with larger farms, given that smaller farms are related to more humanizing production processes (e.g., more handwork on smaller farms) [26, 27]. The following hypothesis (H1b) is proposed: The negative effect of large farming systems compared with smaller ones on the WTB products will be relatively stronger than the positive effect of digital farming technologies on the WTB products.

Industrialized countries have an increasing demand for organic products [28] and policymakers in Europe are pushing towards a higher market share of organic agriculture [29]. In contrast to conventional farming, organic farming prohibits the use of genetically modified organisms (GMOs) and synthetic pesticides, herbicides, and fertilizer in arable farming. Instead, it emphasizes techniques that involve integrated farming systems (e.g., extended crop rotation) [28]. Scholars have found that fear of chemicals and perceived unnaturalness are the main drivers for the higher acceptance of products from organic farms [30]. Thus, the following hypothesis (H1c) is proposed: The negative effect of conventional farming systems compared with organic farming systems on the willingness to buy products is relatively stronger than the effect of digital farming technologies on the WTB of products from conventional farming systems.

### Value orientations shape attitude formation

According to the model developed by [16], values are an important determinant that influences attitude formation through individual perceptions [7]. Values refer to the principles that act to guide behavior [31]. Thus, the commercialization of technologies can be more effective when commonly held values are known [32]. For example, research finds that genetic engineering easily evokes emotions, such as anger, and is usually connected with normative claims [33]. This makes it likely that consumers draw on value orientations when judging genetically engineered foods. Indeed, recent studies show that value orientations related to environmental concerns are more frequently considered in acceptance decisions of technologies in agriculture [34–36].

Along these lines, [37] and [19] proposed that three types of value orientations have an impact on attitude formation, namely altruistic, biospheric, and egoistic concerns. Altruistic concerns provide information on how others, such as farmers, are supported. Biospheric concerns provide information on how the environment is protected. Finally, egoistic concerns cater to the consumers themselves, e.g., through health or other welfare benefits. While altruistic, biospheric, and egoistic concerns are found to impact acceptance [38], the effect of each of these three value orientations on acceptance might differ. Indeed, for sustainable technologies [39] and particular late adopters of those technologies [40], consumer acceptance is mainly driven by egoistic concerns, while biospheric concerns only matter when egoistic concerns are met. Building on this previous research, it is proposed that providing information tailored to the three value orientations (altruistic, egoistic, and biospheric) based on the model of environmental concerns [19] increases the acceptance of DA. Consequently, this study tests the following hypothesis (H2): Environmental benefits of digital farming technologies for farmers, the environment, and oneself will have positive effects on the WTB products from farming systems that utilize DA.

While [16] assume that consumer characteristics, such as moral values, influence the acceptance of agri-food technologies, the formation of attitudes occurs through the perceptions of an individual. In particular, attitudes towards technology are found to influence how individuals perceive technical features of agri-food technologies [41, 42]. For instance, technophilia (technophobia) is a major determinant of the acceptance of food technologies [43, 44] and is especially salient in the early stages of technology diffusion [40]. Applying this notion to the model of consumer acceptance of food technologies developed by [16], attitudes towards technology might influence how individuals perceive environmental concerns and thus shape their evaluation of DA. People with positive attitudes towards technologies likely perceive the environmental concerns of DA as more beneficial than people with negative attitudes towards technology. Therefore, the following hypothesis (H3) is proposed: The stronger general attitudes toward technology are by respondents, the greater the effect of stating environmental arguments will be on respondents’ WTB products from farming systems utilizing DA.

## Methods

### Sample

To investigate the role of mission statements and environmental concerns on consumer’s WTB, this study draws on a sample of German citizens recruited online. In total, 2,299 participants took part in the survey from June to July 2021. Participants were randomly assigned to provide answers to one of eight questionnaires. To ensure the representativeness of each questionnaire, quotas were set according to the distribution of German adults regarding sex, age (older than 15 years), and federal state [45]. The comparison in Table 1 shows that the sample is representative regarding the selected socio-demographics, although respondents between 18 and 24 years of age are slightly overrepresented in the sample, and those 65 years of age or older are slightly underrepresented.

**Table 1:** Minimum and maximum values for respondents’ sex, age, and federal state in the eight questionnaires compared with the German average distribution.

	Minimum	Maximum	Pooled sample	German average
N	248	258	2,020	N/A
Male	48.6%	52.0%	50.5%	49.5%
Female	48.0%	51.4%	49.5%	50.5%
15 to under 18 years	1.6%	3.5%	2.3%	3.2%
18 to under 25 years	10.2%	15.7%	12.3%	8.6%
25 to under 30 years	5.1%	10.4%	7.9%	6.9%
30 to under 40 years	13.9%	19.3%	16.5%	15.2%
40 to under 50 years	12.8%	19.6%	16.8%	14.1%
50 to under 65 years	21.6%	31.8%	26.1%	26.7%
65 years and older	16.1%	20.8%	18.0%	25.5%
Baden-Wuerttemberg	13.7%	19.6%	12.9%	13.4%
Bavaria	14.2%	16.7%	15.7%	15.8%
Berlin	4.7%	4.8%	4.8%	4.4%
Brandenburg	2.0%	3.1%	2.6%	3.0%
Bremen	0.4%	1.2%	1.0%	0.8%
Hamburg	2.3%	2.8%	2.4%	2.2%
Hesse	7.4%	7.9%	7.6%	7.6%
Mecklenburg-Western Pomerania	1.2%	2.0%	1.8%	1.9%
Lower Saxony	8.1%	10.0%	9.3%	9.6%
North Rhine-Westphalia	22.1%	23.0%	22.6%	21.6%
Rhineland-Palatinate	3.2%	5.2%	4.7%	4.9%
Saarland	0.4%	1.2%	1.0%	1.2%
Saxony	4.4%	5.2%	5.0%	4.9%
Saxony-Anhalt	2.0%	2.8%	2.5%	2.6%
Schleswig-Holstein	3.5%	3.6%	3.6%	3.5%
Thuringia	2.0%	2.8%	2.6%	2.5%

Source: Own data and Federal Statistical Office (2020)

A quality check was included to remove participants with a low attention span during the survey by adding a question with an obvious answer: “Please choose the option ‘Disagree’”. After excluding 278 participants, a final sample size of 2,020 participants was obtained. The total sample consists of 50.5% men. The average age of respondents is 45.5 years (from 15 to 74 years of age), and 22.2% have a university degree.

### Study design

**Fig 1:**
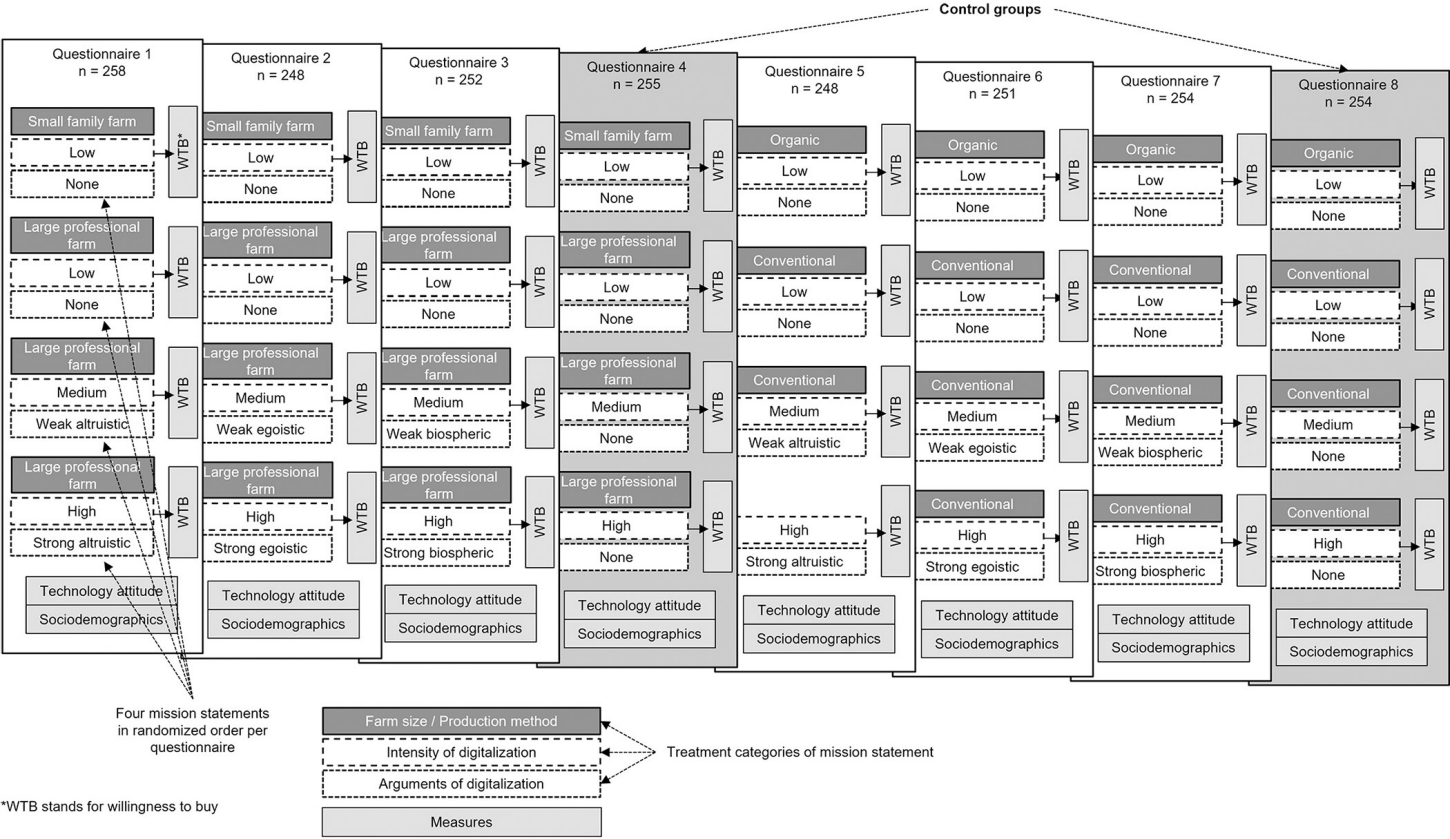
Questionnaire design.

A repeated measure experimental design was used to compare the effects of DA presented within-subject and the effects of environmental arguments presented between-subject on the WTB food produced by different farming systems (see Fig 1). The within-subject compares the following four farming systems: low intensity of digitalization of a small family farm or organic farm, low intensity of digitalization of a large professional farm or conventional farm, medium intensity of digitalization of a large or conventional farm, and high intensity of digitalization of a large or conventional farm. The four environmental arguments are: no argument and altruistic, egoistic, and biospheric arguments. The questionnaires differ by their treatments in the mission statements. Each mission statement consists of three treatment categories: 1) farm size/farm type; 2) intensity of digitalization; 3) arguments of digitalization.

Before the survey was carried out, the farming systems and environmental arguments were pretested on 144 and 92 students, respectively, to ensure the understandability of the mission statements. WTB based on each mission statement was assessed for participants in each group based on a randomized order of the four mission statements. In addition, the respondents’ general attitudes towards technologies and sociodemographic characteristics were examined.

**Fig 2:**
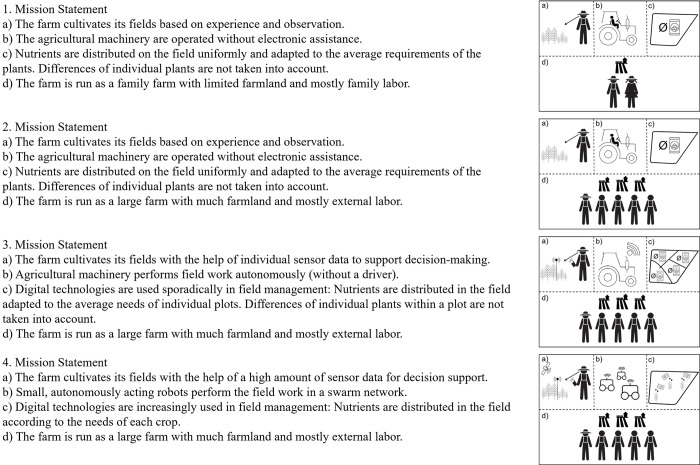
Mission statements with different farm sizes and altruistic arguments within subject.

Fig 2 shows the four mission statements with varying farm sizes. Each mission statement includes four pictures accompanied by a brief text describing the illustrated pictures. Three pictures describe the intensity of DA used on the farm. In line with previous literature, DA fundamentally improves arable agriculture in three main ways: 1) DA fosters agronomic decision-making [46]; 2) DA improves job execution through a higher degree of job automation [47]; and c) DA leads to higher precision of input applications to the sub-field [48] or single plant variability [49]. The three intensity levels of DA are described through three farming systems using different digital technologies and are defined as follows: First, non-digitalized agronomic decision making, manual machine operation and no sub-field precision of input application. Second, digitally assisted agronomic decision making, autonomous operation of equipment and site-specific precision of input application. Third, highly digitally assisted agronomic decision making, field operations by small autonomous robots and plant-level precision of input application. Furthermore, the fourth picture (d) illustrates the farm size (small family farm or large professional farm) or production method (organic crop production or conventional crop production) since both are found as affecting acceptance (cf. [6] for an overview). The first mission statement contains a small family farm (or an organic farm) with a low intensity of DA. The other mission statements contain a professional farm (or a conventional farm) with either a low, medium, or high intensity of digitalization. All mission statements were shown in random order. We are aware that the type of organizations (e.g., family-run farm) is not necessarily related to the size of the farm (e.g., size of cultivated land), although family farms–implying that they are organized as individual entrepreneurs or partnerships–are, on average, much smaller than professional farms that operate as legal entities [50]. However, this operationalization likely depicts society’s perception of agricultural farms for two reasons. First, the differentiation between small family farms and large professional farms is frequently used in public discussions and by policymakers in Germany (e.g., through corporations under public law) [51]. Second, since the foundation of the Common Agricultural Policy in Europe, one of its main goals is to protect small family farm structures [52].

Further, for six of the eight questionnaires, arguments of the farming system depicted in the mission statement were added to the text description. Two questionnaires (one for farm size and the other for production method) contain no details about advantages and serve as control groups. The arguments vary in their strength concerning the intensity of DA. For farming systems where a medium intensity of DA is embedded, the arguments stated that DA cause “some improvement,” while the arguments of farming systems where a high intensity of DA is embedded cause “strong improvement.” The arguments included biospheric, egoistic, or altruistic benefits that result from DA. For example, biospheric arguments of DA were operationalized as a “reduction of environmental pollution through reduced use of fertilizer and pesticides to some (or a great) extent” and a “reduction of harmful soil compaction through fewer field crossings and smaller (lighter) tractors to some (or a great) extent.” Egoistic arguments contain “reducing health-damaging residues of pesticides in food through reduction of pesticide use to some (or a great) extent” and “improvement of transparency in production through digital traceability to some (or a great) extent.” “Reduction of the workload through more comfort to some (or a great) extent” and “reduction of risk in crop production through the substantial decline of yield caused by draughts to some (or a great) extent” were altruistic arguments.

### Measures

All constructs of the dependent and independent variables included in the questionnaire were measured on 5-point Likert scales, anchored with strongly disagree [1] and strongly agree [5]. To collect data on the dependent variables, the respondents were asked to assess their WTB products produced by the example farm described in the mission statement. WTB was measured with the single variable “I would buy food produced by this farm,” which is a well-established approach in the literature to assess acceptance (cf. [53]). The potential moderator variable was attitudes towards technologies. Attitudes towards technologies were measured using two items that were computed by calculating the mean of both items. The items were taken from [18] (“technology is a danger for humans and their environment” and “technology makes life more comfortable”).

### Statistical analysis

To evaluate H1, within repeated-measures analyses of variances (ANOVAs) were employed, examining the differences between farming systems (farm size, production method, and intensity of DA used) in consumers’ WTB. Further, two between-subject ANOVAs were run for farming systems that employed medium and high intensities of DA to investigate H2 regarding the effects of environmental arguments (no, altruistic, egoistic, and biospheric arguments) on consumers’ WTB food produced by these farming systems. Finally, for H3, the interaction effects of general attitudes towards technology were tested through four separated ANOVAs for environmental arguments.

## Results

Table 2 shows the WTB food produced by different farming systems. Within-subject repeated measurement ANOVAs yielded a significant effect for farm size, namely Greenhouse-Geisser F(1012, 1) = 293.4 with p < .001. Results of the Bonferroni post-hoc test revealed that WTB is significantly higher for food produced by small family farms (M = 4.13, SE = 0.88) compared with large professional farms (M = 3.59, SE = 0.98). Furthermore, the results of an ANOVA showed a significant effect for production method, namely Greenhouse-Geisser F(1006, 1) = 315.76 with p < .001. Results of the Bonferroni post-hoc test revealed that WTB is significantly higher for food produced by organic farms (M = 4.06, SE = 0.92) compared with conventional farms (M = 3.33, SE = 1.09). Finally, results of an ANOVA showed a significant effect for DA when farm size and production method questionnaires were pooled, namely Greenhouse-Geisser F(2.74, 5530.29) = 351.51 with p < .001. According to the Bonferroni post-hoc test, WTB was significantly higher for food produced by small family farms/organic farms (M = 4.09, SE = 0.9) in comparison with large professional farms/conventional farms where low (M = 3.46, SE = 1.05), medium (M = 3.34, SE = 1.08), and high (M = 3.39, SE = 1.13) intensities of DA were incorporated. Thus, the test supported H1b and H1c, suggesting that WTB of food produced by large professional farms and conventional farms does not reach the level of small family farms (H1b) or organic farms (H1c) when DA was incorporated.

**Table 2:** Willingness to buy food based on different farming systems.

Condition	WTB		
Only farm size questionnaires	M	SD	N
Small family farm with a low intensity of digital technologies	4.13^a^	0.88	1,013
Large professional farm with a low intensity of digital technologies	3.59^b^	0.98	
Condition	WTB		
Only production method questionnaires	M	SD	N
Organic farm with a low intensity of digital technologies	4.06^a^	0.92	1,007
Conventional farm with a low intensity of digital technologies	3.33^b^	1.09	
Condition	WTB		
Pooled questionnaires (farm size and production method)	M	SD	N
Small family farm/organic farm with a low intensity of digital technologies	4.09^a^	0.9	2,020
Large professional farm/conventional farm with a low intensity of digital technologies	3.46^b^	1.05	
Large professional farm/conventional farm with a medium intensity of digital technologies	3.34^c^	1.08	
Large professional farm/conventional farm with a high intensity of digital technologies	3.39^bc^	1.13	

Note: Farm properties were within-subject conditions from repeated measures (ANOVA and Bonferroni post-hoc tests). Different superscripts within column indicate significant difference at p < .05. M stands for mean. SD stands for standard deviation.

WTB of food produced by large professional farms/conventional farms was significantly higher when a low intensity of DA was incorporated into the illustrated farming system compared with a medium intensity of DA. However, WTB was not significantly different between large professional farms/conventional farms incorporating either low intensity or high intensity of DA. Therefore, there is no support for H1a: WTB of food produced by a farming system actually decreases from a low to medium intensity of DA, while there might be no difference between medium and high intensities of DA.

Between-subjects one-way ANOVAs were used to compare the effects of environmental arguments (no arguments, altruistic arguments, egoistic arguments, and biospheric arguments) added to mission statements of large professional farms/conventional farms with medium and high intensities of DA on the WTB food produced by these farming systems (see Table 3). The ANOVA testing WTB food produced by large professional farms/conventional farms with a medium intensity of DA showed no significant effect between environmental arguments, namely *Greenhouse-Geisser F*(3, 2016) = 1.96 with p = .64. Results of Tukey’s honestly significant difference (HSD) post-hoc test showed that adding environmental arguments does not significantly increase the WTB food produced by large professional farms/conventional farms where a medium intensity of DA was incorporated. Furthermore, results of the ANOVA showed no significant effect for large professional farms/conventional farms with a high intensity of DA, namely *Greenhouse-Geisser F*(3, 2016) = 2.29 with p = .08. According to Tukey’s HSD, WTB food produced by large professional farms/conventional farms with a high intensity of DA was significant higher when biospheric arguments were stated (M = 3.47, SE = 1.03) in comparison with no arguments (M = 3.28, SE = 1.16), but not significantly different for altruistic (M = 3.4, SE = 1.1) and egoistic (M = 3.3, SE = 1.14) arguments. Therefore, there is some support for H2: Environmental arguments can increase WTB food produced by farming systems that incorporate DA. However, the effects on WTB vary depending on whether these arguments involve altruistic, egoistic, or biospheric concerns.

**Table 3:** Willingness to buy food produced by farms based on environmental arguments.

	Medium intensity of digital technologies		High intensity of digital technologies		N
Condition	Willingness to buy		Willingness to buy		
Environmental arguments	M	SD	M	SD	
None	3.31^a^	1.09	3.28^a^	1.16	509
Altruistic	3.38^a^	1.07	3.4^ab^	1.1	506
Egoistic	3.3^a^	1.14	3.4^ab^	1.21	499
Biospheric	3.35^a^	0.99	3.47^bc^	1.03	506

Note: Environmental arguments were between-subject conditions from repeated measures ANOVA and Tukey HSD post-hoc tests. Different superscripts within column indicate significant difference at p < .05. M stands for mean. SD stands for standard deviation.

**Fig 3:**
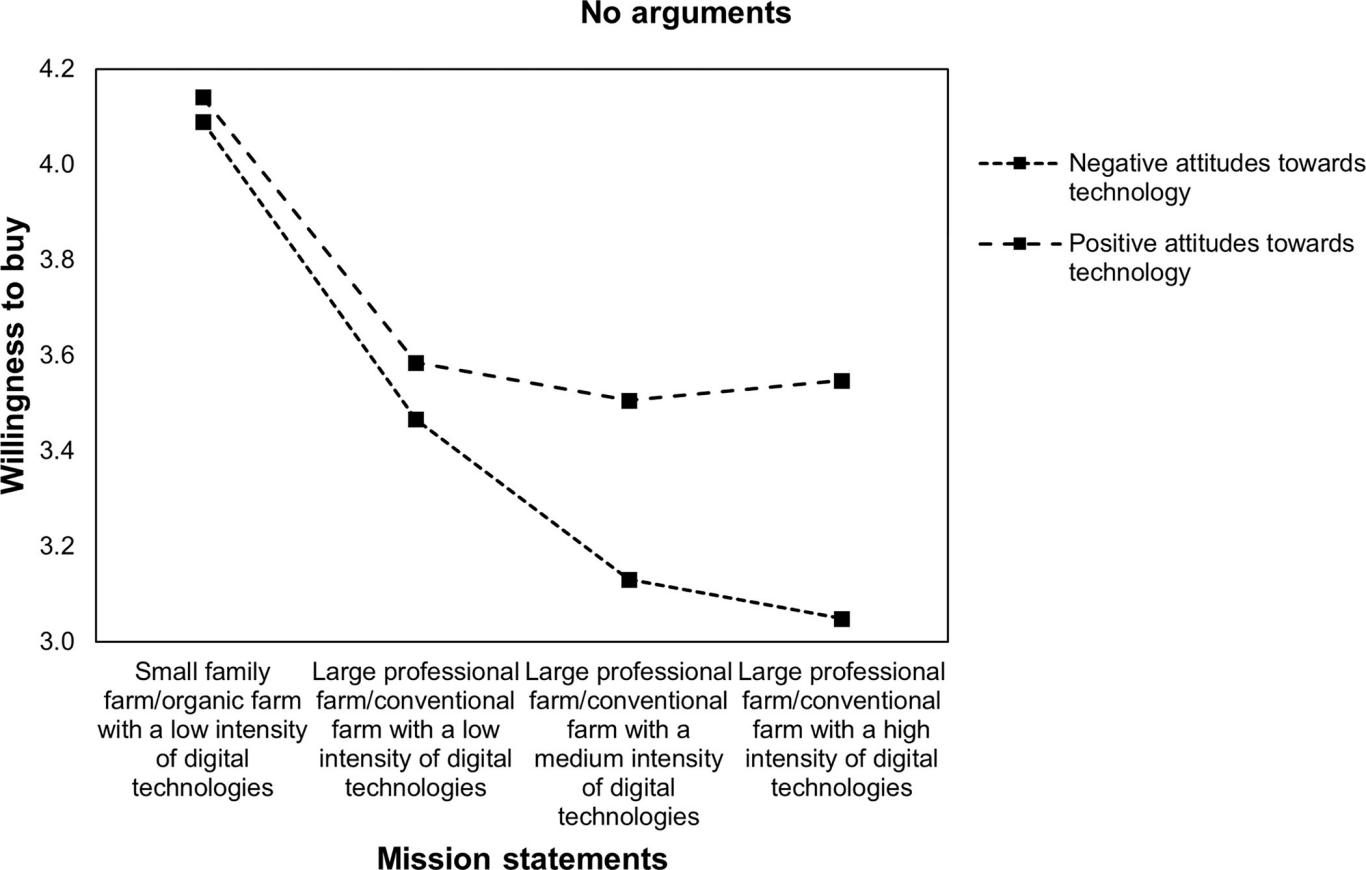
Profile plot of WTB food and attitudes towards technology without environmental arguments.

**Fig 4:**
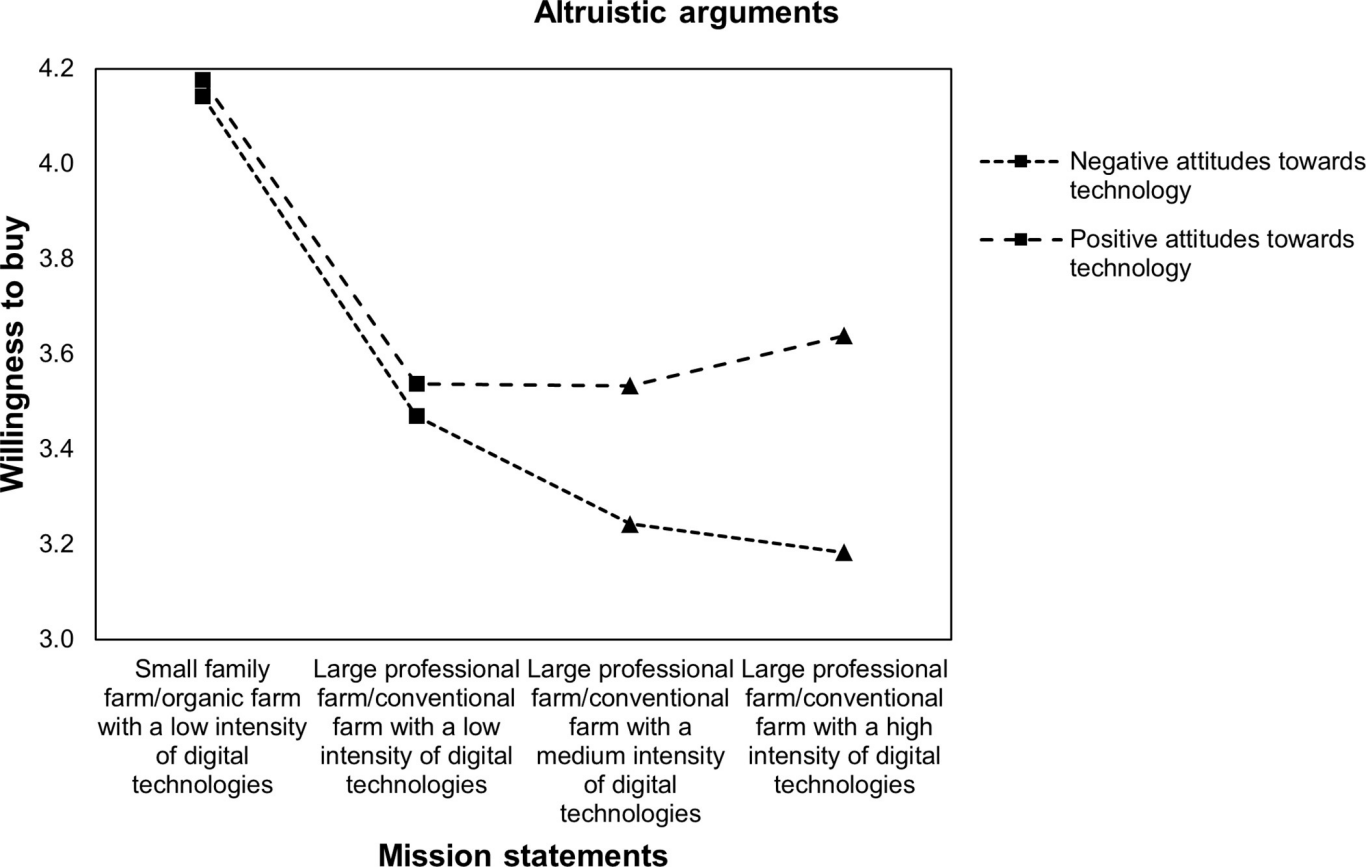
Profile plot of WTB food and attitudes towards technology with altruistic arguments.

**Fig 5:**
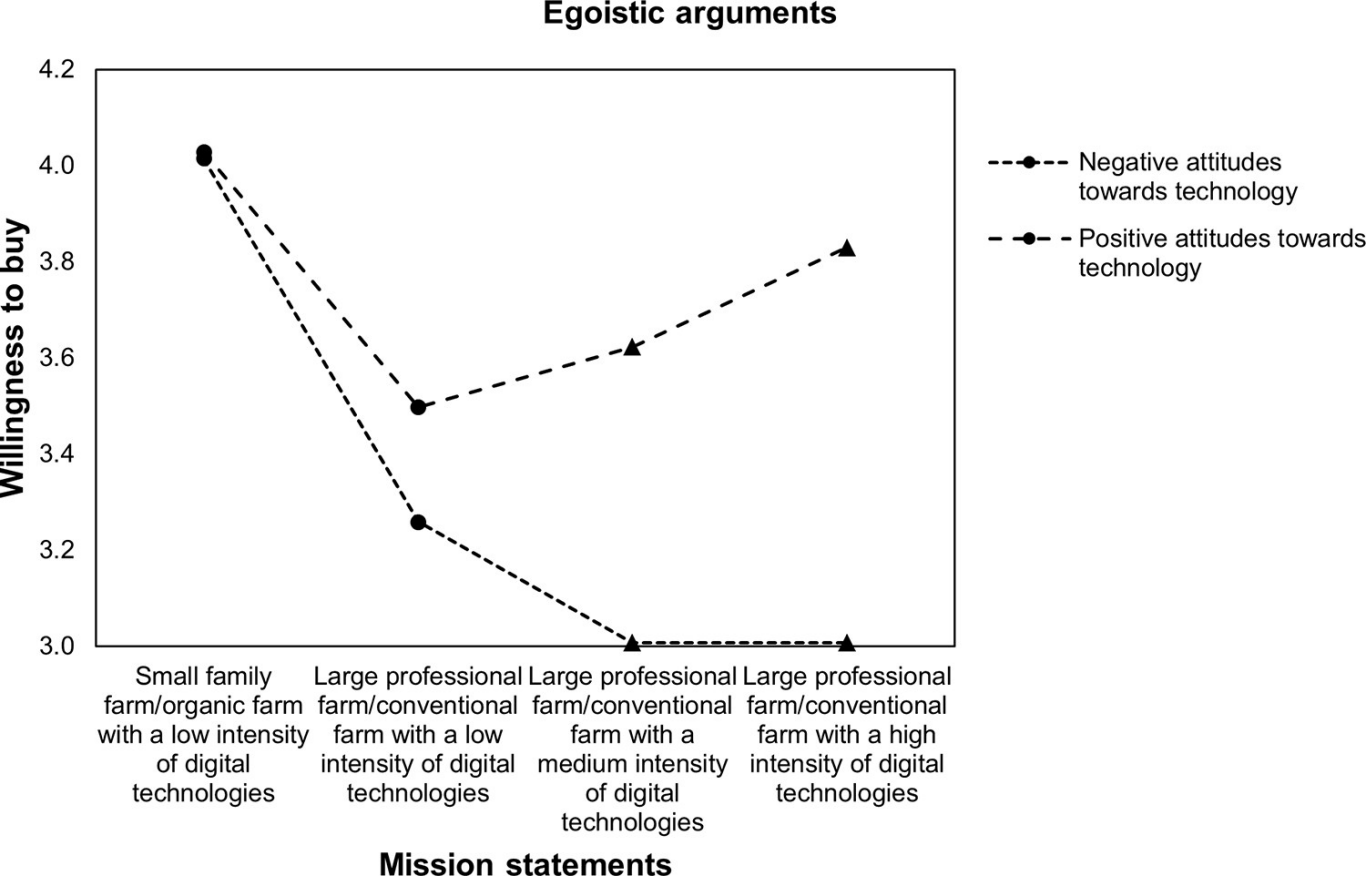
Profile plot of WTB food and attitudes towards technology with egoistic arguments.

**Fig 6:**
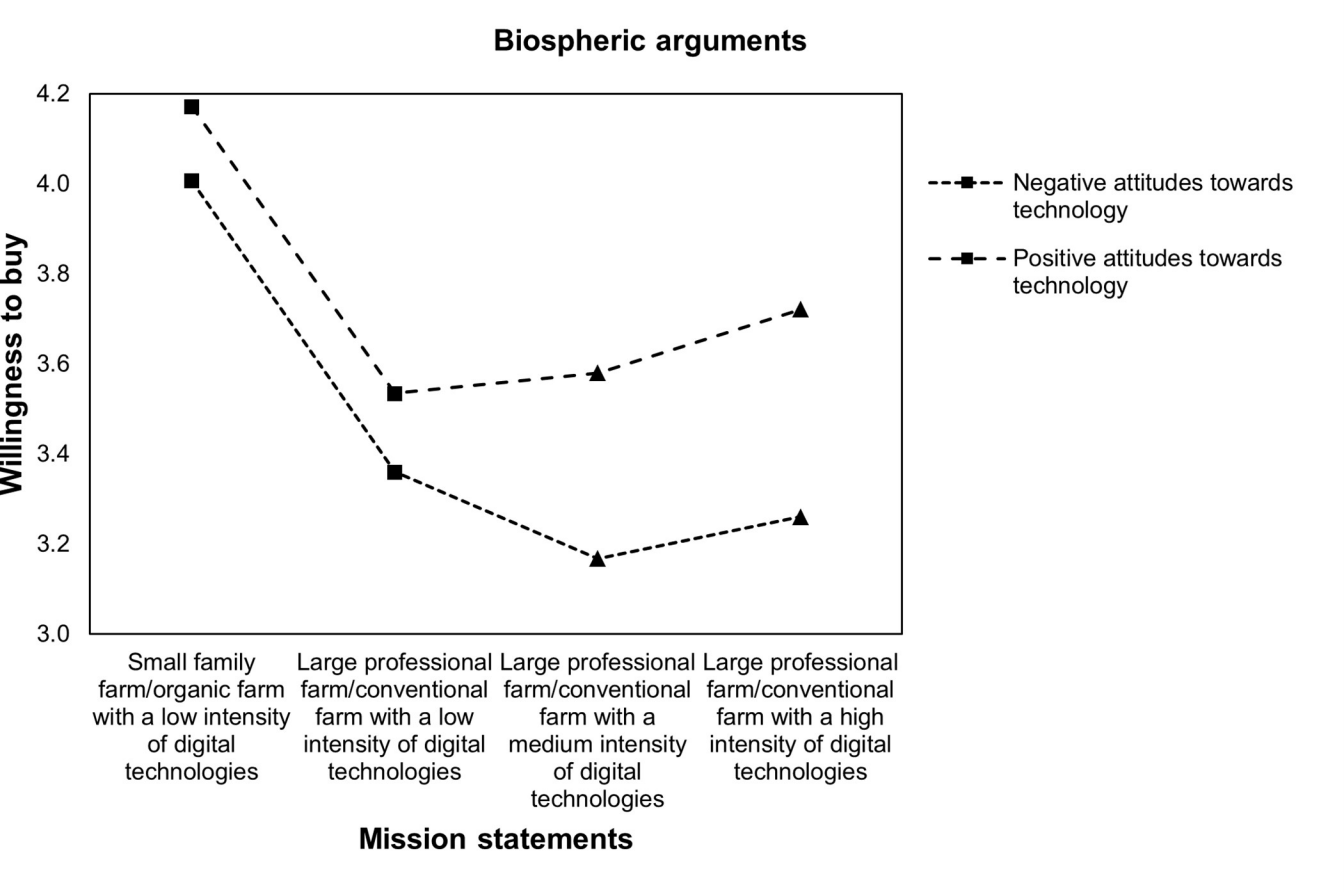
Profile plot of WTB food and attitudes towards technology with biospheric arguments.

Finally, the sample was split by respondents’ general attitudes towards technology to assess if relationships between the farming system and WTB vary between different environmental arguments and among respondents with positive and negative attitudes towards technology. Figs 3–6 display the relationship between WTB and different farming systems in the presence of positive (above mean) and negative (below mean) attitudes towards technology. Therefore, within-subject 4 (farming system: comparing small family farm/organic farm with a low intensity of DA, large professional farm/conventional farm with a low intensity of DA, large professional farm/conventional farm with a medium intensity of DA, and large professional farm/conventional farm with a high intensity of DA) x 2 (technology attitudes: comparing negative and positive) ANOVAs were conducted. The results show significant interactions between attitudes towards technology and WTB, namely *Greenhouse-Geisser F*(2.67, 1353,03) = 7.85 with p < .001 when no environmental concerns are stated. Fig 3 illustrates that WTB is similar for a large professional farm/conventional farm with low (M = 3.59, SE = 1.01), medium (M = 3.51, SE = 1.06), and high intensities of DA (M = 3.55, SE = 1.11) among respondents with positive attitudes towards technology. The results also show that WTB decreases for a large professional farm/conventional farm with a low intensity of DA (M = 3.47, SE = 1) compared with a farm with a medium intensity of DA (M = 3.13, SE = 1.1). Moreover, WTB is lowest for a large professional farm/conventional farm with a high intensity of DA (M = 3.05, SE = 1.15).

Moreover, results of ANOVA showed significant interactions between attitudes towards technology and WTB in the presence of altruistic arguments, namely Greenhouse-Geisser F(2.84, 1433.06) = 7.27 with p < 0.001. Fig 4 illustrates that for respondents with positive attitudes towards technology, WTB remains equal for large professional farms/conventional farms between a low intensity of DA (M = 3.54, SE = 1.08) and medium intensity of DA when altruistic arguments are added (M = 3.53, SE = 1.1) and slightly increases for a high intensity of DA in the presence of altruistic arguments (M = 3.64, SE = 1.1). In contrast, when supported by altruistic arguments, respondents with negative attitudes towards technology had a higher WTB food produced by a large professional farm/conventional farm with a low intensity of DA (M = 3.47, SE = 1.06) compared with farms with medium (M = 3.24, SE = 1.03) or high intensities of DA (M = 3.18, SE = 1.05).

The results from the ANOVA revealed significant interactions between attitudes towards technology and WTB given egoistic arguments, namely *Greenhouse-Geisser F*(2.73, 1354,2) = 21.69 with p < .001. Looking at the interaction graph in Fig 5, this effect reflects that when mission statements were accompanied by egoistic arguments, positive attitudes toward technology increase WTB as the intensity of DA increases, namely from low (M = 3.5, SE = 1.12) to medium (M = 3.62, SE = 1.07) and to high intensities of DA (M = 3.83, SE = 1.06). For respondents with negative attitudes towards technology, medium (M = 3.01, SE = 1.13) and high intensities of DA (M = 3.01, SE = 1.2) accompanied by egoistic arguments resulted in lower acceptance compared with large professional farms/conventional farms with a low intensity of DA (M = 3.26, SE = 1.58).

Finally, there was a significant interaction effect between attitudes towards technology, WTB, and biospheric arguments between different farming systems, namely *Greenhouse-Geisser F* (2.81, 1416.59) = 4.66 with p = .004. The interaction graph in Fig 6 shows that WTB remains equal between large professional farms/conventional farms with a low intensity of DA (M = 3.54, SE = 1.06) and those with a medium intensity given biospheric arguments (M = 3.58, SE = 0.98). Moreover, WTB slightly increases for farms with a high intensity of DA given biospheric arguments (M = 3.72, SE = 1.02). For respondents with negative attitudes towards technology, WTB only slightly decreases for large professional farms/conventional farms with medium (M = 3.17, SE = 0.97) and a high intensity of DA (M = 3.26, SE = 0.99) given biospheric arguments compared with large professional farms/conventional farms with a low intensity of DA (M = 3.36, SE = 0.97).

Therefore, the results support Hypothesis 3: Both, respondents with positive and negative attitudes towards technologies are receptive to environmental concerns. However, attitudes towards technologies seem to shift the reference point of acceptance and the responsiveness to different arguments. That is, DA is neutral and not stigmatized for individuals with positive attitudes towards technologies (no difference between large professional farms/conventional farms with low, medium, and high intensities of DA). Instead, DA has a negative connotation among individuals with negative attitudes towards technologies. Furthermore, respondents with negative attitudes towards technology seem to be most receptive to biospheric arguments, while those with positive attitudes are most receptive to egoistic arguments.

## Discussion

This study sought to investigate the influence DA has on the acceptance of farming systems. Global agriculture has attained efficient food production through intensification and consolidation of farms, but at the same time created environmental damage and a lack of public acceptance. Thus, DA has been suggested as a potential solution to reconcile environmental sustainability and yield increase. However, for the development of new agricultural systems, public acceptance is crucial [54]. While politics [50] and the private sector [55, 56] assume that DA, as a moderator between efficiency and environmental friendliness [11], can lead to an increased public acceptance, current literature lacks empirical evidence.

In the present study, a general decrease in WTB food produced by large professional farms/conventional farms compared with small family farms/organic farms was found. Moreover, WTB further decreases for large professional farms/conventional farms when DA was embedded into mission statements but increases for large professional farms/conventional farms with a high intensity of DA when environmental concerns are stated. However, the results found a positive moderation effect on WTB from increasing DA intensities among individuals with positive attitudes towards technologies. This effect is highest for egoistic arguments for DA. The results also showed a negative moderation effect among individuals with negative attitudes towards technologies. This negative effect is lowest for biospheric arguments. Furthermore, realized increases in WTB food produced by large professional farms and conventional farms with DA are still lower than that for small family farms and organic farms without DA.

The study contributes to a better understanding of the impact of DA on the acceptance (i.e., WTB) of present and future farming systems, which is an important predictor of the diffusion of digital technologies. In particular, this paper contributes to the literature on the acceptance of food technologies by revealing: (1) DA harms public acceptance of farming systems; (2) the negative effect of DA on WTB can be reversed by including environmental arguments; and (3) the effects of other farm characteristics, namely small family farm vs. large professional farm and organic production versus conventional production, are relatively stronger than those of DA.

This paper refines the perspective on drivers of DA acceptance to better understand variance in public acceptance among present and future farming systems where different intensities of DA are incorporated [14]. In particular, technophilic people who are more open to new technologies are more likely to support farming systems with a higher intensity of DA, while technophobic people who are more skeptical towards new technologies are more likely to reject farming systems with a higher intensity of DA. Thus, it is emphasized that DA has no guarantee for increasing public acceptance, but it can–in the case of proper communication–foster acceptance of future farming systems since the perception of it depends strongly on individuals’ general attitudes towards technology. These insights contribute to the ongoing discussion on the mechanisms underlying individuals’ buying behavior of food from different agricultural production methods (e.g., organic vs. conventional farming [57]) and produced by different technologies (e.g., nanotechnology [53]). Individuals’ attitudes restrict the assimilation of information and rational consideration, and can therefore pre-determine the success of communication strategies.

Interestingly, the results (in Table 1) showed not only that acceptance is higher for organic farms and small family farms compared with conventional farms and large professional farms, respectively, but also revealed that embedding DA cannot reverse this trend. Although DA can enable more sustainable farming systems, the general public is not seeing this as beneficial. Many citizens seem to have a rather fixed opinion on what “good” or “bad” farming systems are like, which might be rooted in moral values. One reason for this situation might be that the public discussion around agriculture has been focused on the process of food production, the “what” and “how” of farming. That is, production measures related to credence attributes, such as perceived naturalness of food production [7] or whether the technology applied is deemed as traditional [58], are considered relatively more important in public discussions than the actual environmental and social outcomes of various farming systems. As such, the recent trajectory of DA—namely technologies that enable production methods that rely less on human intervention (e.g., weed control through autonomous robots)–likely fosters environmental and social outcomes at the cost of decreasing perceived natural processing. Therefore, as long as there is not a stronger focus on actual outcomes in the public discussion on the future of agriculture, DA is likely to struggle to show the desired positive effect on public acceptance [59], especially among those who generally hold negative attitudes towards technology. Furthermore, such a moral judgment might hamper research and development (R&D) investments for technologies that reduce human intervention [60] yet foster SI of farming systems.

Additionally, the results (in Figs 3–6) illustrate that the positive effect of environmental arguments on the perception of DA varies strongly among respondents with positive and negative attitudes towards technologies. While acceptance of DA was highest among respondents with positive attitudes towards technologies when arguments for oneself were stated, respondents with negative attitudes towards technologies showed the highest rates of acceptance for environmental arguments. At the same time, the model of environmental concerns [19] for farming systems and DA was confirmed. Value considerations are indeed taken into account when assessing farming systems and DA. Moreover, depending on individual differences, the three value orientations proposed by [19], namely egoistic, biospheric, and altruistic, have different impacts on WTB. These findings refine the theoretical framework of [16] on the acceptance of technology-based food innovations. Technical features, such as environmental concerns, lead to acceptance through their perception. Indeed, the results in this paper showed that attitudes towards technologies are one important determinant that shapes how individuals evaluate DA.

In addition to the above contributions to the literature, the findings are highly relevant to policymakers and the private sector. Fostering SI is a crucial aspect to feed an increasing world population while reducing environmental impacts. The results show that society places a greater emphasis on the process of food production than on actual environmental outcomes. Policymakers and the private sector are well-advised to focus communication strategies more on outcome-based judgments of food production to reduce the gap between technological advances and society.

### Limitations

Several limitations of the study suggest promising avenues for future research. The study design enabled digital technologies to be embedded into holistic arable farming systems. The described mission statements contained pictures and descriptions of the respective farming systems. Although the comprehensibility of the mission statements was carefully assessed through several pretests, some respondents may have misunderstood them. This is particularly the case in regard to the effect of environmental concerns provided in the written statements below the description of the mission statements. An optimal approach would involve validation of whether respondents fully understood the mission statements and respective environmental concerns. Furthermore, the content of the environmental concerns may bias results. Although the arguments were created carefully, one could argue that other arguments regarding environmental concerns might affect the relationship between environmental concerns and acceptance. Therefore, it would be interesting to assess whether and to what extent different environmental concerns affect the results.

Second, the mission statements were conceptualized through the intensity of DA based on decision support, automation, and precision. This approach allowed rich empirical data to be gathered on the acceptance of DA embedded in holistic farming systems. However, an optimal approach would involve the assessment of the validity of DA, such as by following rigorous scale development procedures [61]. Furthermore, respondents’ relative importance on each aspect of DA cannot be assessed. For example, autonomous machines–incorporated in the mission statements with high intensities of DA–may trigger more negative connotations, while other aspects, such as the degree of application precision, may trigger more positive perceptions (e.g., through increased efficiency); however, this has yet to be proven. Although the assessment of holistic farming systems likely yields more accurate results regarding the effect of DA on acceptance, examining the acceptance of single technologies might advance research on responsible innovations of these particular technologies [62, 63]. Moreover, since the study focused on examining the potential of DA to increase intensive farming systems, the effect of DA utilized in small family farms and organic farms was not assessed. For future research, it would be insightful to examine whether the obtained results of DA also hold for these farms.

Third, the applied cross-sectional study design might weaken the results. Although the literature suggests that environmental concerns and their weighing through personal cognition is likely to influence individuals’ acceptance, temporal affective states might also influence how individuals perceive acceptance, which was not captured through the study design. Hence, scholars might be able to replicate the results by applying other, more longitudinal research designs. Further, we cannot rule out potential bias resulting from the assessment of WTB through a Likert scale. Although this procedure is in line with existing research (cf. [53], respondents’ buying behavior might differ from what they state in questionnaires due to social desirability. Moreover, as the respondents were recruited via an online panel provider, we cannot rule out selection bias regarding digital competencies (i.e., only individuals less averse to technologies participate in online surveys).

## Conclusions

The present study tests the co-occurrence of digital technologies embedded into holistic farming systems and their environmental arguments on the WTB food produced by these farms among German adults. By applying a 4 (farming system: low intensity of digitalization for small or organic farms and low, medium, and high intensity of digitalization for large or conventional farms) x 4 (environmental arguments: none, altruistic, egoistic, biospheric) repeated measure research design that simplifies the understanding of complex technologies incorporated in future farming systems through visualized mission statements, the study revealed that WTB decreases for food produced by large professional farms/conventional farms compared with small family farms/organic farms. Moreover, WTB further decreased for large professional farms/conventional farms when DA was incorporated. However, in the presence of written statements addressing environmental concerns, WTB increases for food produced with a high intensity of DA to the level of the WTB food produced from farming systems with a low intensity of DA. Furthermore, general attitudes towards technologies leverage respondents’ receptiveness to environmental concerns. That is, the moderation effect is positive for technophile respondents’ WTB and highest for egoistic arguments of DA, whereas it is negative for technophobia respondents’ WTB and is lowest for biospheric arguments of DA. These results contribute to a better understanding of the impact of DA on the acceptance (i.e., WTB).

Moreover, the results confirm the model of environmental concerns [19] for farming systems and DA: value considerations are indeed taken into account when assessing farming systems and DA. Moreover, depending on individual differences, the three value orientations proposed by [19], namely egoistic, biospheric, and altruistic, have different impacts on WTB. These findings refine the theoretical framework of [16] on the acceptance of technology-based food innovations.

Furthermore, the results also refine the perspective on drivers of DA acceptance by showing that technophilic people who are more open to new technologies are more likely to support farming systems with a higher intensity of DA, while technophobic people who are more skeptical towards new technologies are more likely to reject farming systems with a higher intensity of DA. Thus, it is emphasized that DA has no guarantee for increasing public acceptance, but it can–in the case of proper communication–foster acceptance of future farming systems since the perception of it depends strongly on individuals’ general attitudes towards technology.

The findings suggest to policymakers and the private sector to that such communication strategies should take into account the preferences of target groups, and focus more on outcome-based judgments of food production to reduce the gap between technological advances and society.

## References

[1] Pingali PL. Green Revolution: Impacts, limits, and the path ahead. Proceedings of the National Academy of Sciences. 2012;109(31):12302–8. 10.1073/pnas.0912953109 PMC3411969 22826253

[2] Godfray HCJ, Beddington JR, Crute IR, Haddad L, Lawrence D, Muir JF, et al. Food security: The challenge of feeding 9 billion people. Science. 2010;327(5967):812–8. doi: 10.1126/science.1185383 20110467

[3] United Nations. World Population Prospects 2019 Highlights. New York; 2019.

[4] Hallmann CA, Sorg M, Jongejans E, Siepel H, Hofland N, Schwan H, et al. More than 75 percent decline over 27 years in total flying insect biomass in protected areas. PLOS ONE. 2017;12(10):e0185809–e. doi: 10.1371/journal.pone.0185809 29045418 PMC5646769

[5] Weis T. The Accelerating Biophysical Contradictions of Industrial Capitalist Agriculture. Journal of Agrarian Change. 2010;10(3):315–41.

[6] Etale A, Siegrist M. Food processing and perceived naturalness: Is it more natural or just more traditional? Food Quality and Preference. 2021;94:104323–.

[7] Román S, Sánchez-Siles LM, Siegrist M. The importance of food naturalness for consumers: Results of a systematic review. Trends in Food Science & Technology. 2017;67:44–57.

[8] Meulen HABvd Dolman MA, Jager JH Venema GS. The impact of farm size on sustainability of dutch dairy farms. International Journal of Agricultural Management. 2014;3(2):119–23.

[9] Smith LG, Kirk GJD, Jones PJ, Williams AG. The greenhouse gas impacts of converting food production in England and Wales to organic methods. Nature Communications 2019 10:1. 2019;10(1):1–10. doi: 10.1038/s41467-019-12622-7 31641128 PMC6805889

[10] Garnett T, Appleby MC, Balmford A, Bateman IJ, Benton TG, Bloomer P, et al. Sustainable intensification in agriculture: Premises and policies. Science. 2013;341(6141):33–4. 23828927 10.1126/science.1234485

[11] Basso B, Antle J. Digital agriculture to design sustainable agricultural systems. Nature Sustainability. 2020;3(4):254–6.

[12] Blok V, Gremmen B. Agricultural Technologies as Living Machines: Toward a Biomimetic Conceptualization of Smart Farming Technologies. Ethics, Policy & Environment. 2018;21(2):246–63.

[13] Gupta N, Fischer ARHH, Frewer LJ. Socio-psychological determinants of public acceptance of technologies: A review. Public understanding of science (Bristol, England). 2012;21(7):782–95. doi: 10.1177/0963662510392485 23832558 PMC3546631

[14] Ofori M, El-Gayar O. Drivers and challenges of precision agriculture: a social media perspective. Precision Agriculture. 2021;22(3):1019–44.

[15] Pfeiffer J, Gabriel A, Gandorfer M. Understanding the public attitudinal acceptance of digital farming technologies: a nationwide survey in Germany. Agriculture and Human Values. 2020;1:3–.

[16] Ronteltap A, van Trijp JCM, Renes RJ, Frewer LJ. Consumer acceptance of technology-based food innovations: Lessons for the future of nutrigenomics. Appetite. 2007;49(1):1–17. doi: 10.1016/j.appet.2007.02.002 17382433

[17] Bearth A, Siegrist M. Are risk or benefit perceptions more important for public acceptance of innovative food technologies: A meta-analysis. Trends in Food Science & Technology. 2016;49:14–23.

[18] Siegrist M, Keller C, Kastenholz H, Frey S, Wiek A. Laypeople’s and Experts’ Perception of Nanotechnology Hazards. Risk Analysis. 2007;27(1):59–69. doi: 10.1111/j.1539-6924.2006.00859.x 17362400

[19] Stern PC, Dietz T, Kalof L. Value Orientations, Gender, and Environmental Concern. Environment and Behavior. 1993;25(5):322–48.

[20] Hoogendoorn G, Sütterlin B, Siegrist M. Tampering with Nature: A Systematic Review. Risk Analysis. 2021;41(1):141–56. 33141501 10.1111/risa.13619

[21] Springmann M, Clark M, Mason-D’Croz D, Wiebe K, Bodirsky BL, Lassaletta L, et al. Options for keeping the food system within environmental limits. Nature. 2018;562(7728):519–25. doi: 10.1038/s41586-018-0594-0 30305731

[22] Commission European. Special Eurobarometer 460—Attitudes towards the impact of digitalisation and automation on daily life. 2017.

[23] Belfrage K, Björklund J, Salomonsson L. Effects of Farm Size and On-Farm Landscape Heterogeneity on Biodiversity—Case Study of Twelve Farms in a Swedish Landscape. Agroecology and Sustainable Food Systems. 2015;39(2):170–88.

[24] Fahrig L, Girard J, Duro D, Pasher J, Smith A, Javorek S, et al. Farmlands with smaller crop fields have higher within-field biodiversity. Agriculture, Ecosystems & Environment. 2015;200:219–34.

[25] Stoate C, Boatman ND, Borralho RJ, Carvalho CR, De Snoo GR, Eden P. Ecological impacts of arable intensification in Europe. Journal of Environmental Management. 2001;63(4):337–65. doi: 10.1006/jema.2001.0473 11826719

[26] Abouab N, Gomez P. Human contact imagined during the production process increases food naturalness perceptions. Appetite. 2015;91:273–7. doi: 10.1016/j.appet.2015.04.002 25862979

[27] Scekic A, Krishna A. Do Firm Cues Impact Product Perceptions? When Small is Natural. Journal of Consumer Psychology. 2021;31(2):350–9.

[28] European Parliament. The EU’s organic food market: facts and rules. 2020.

[29] European Commission. A Farm to Fork Strategy for a fair, healthy and environmentally-friendly food system. 2020.

[30] Saleh R, Bearth A, Siegrist M. How chemophobia affects public acceptance of pesticide use and biotechnology in agriculture. Food Quality and Preference. 2021;91:104197–.

[31] Schwartz SH. Universals in the Content and Structure of Values: Theoretical Advances and Empirical Tests in 20 Countries. Advances in Experimental Social Psychology. 1992;25:1–65.

[32] Groot JIMd, Steg L. Value Orientations to Explain Beliefs Related to Environmental Significant Behavior: How to Measure Egoistic, Altruistic, and Biospheric Value Orientations. Environment and Behavior. 2007;40(3):330–54.

[33] Emnid TNS. Das Image der deutschen Landwirtschaft [The image of German agriculture]. 2012.

[34] Broad GM, Marschall W, Ezzeddine M. Perceptions of high-tech controlled environment agriculture among local food consumers: using interviews to explore sense-making and connections to good food. Agriculture and Human Values. 2021;1:1–17.

[35] Jürkenbeck K, Heumann A, Spiller A. Sustainability Matters: Consumer Acceptance of Different Vertical Farming Systems. Sustainability 2019, Vol 11, Page 4052. 2019;11(15):4052-.

[36] Nielsen T. Consumer Buying Behavior of Genetically Modified Fries in Germany. Journal of Food Products Marketing. 2013;19(1):41–53.

[37] Schultz WP. The structure of environmental concern: Concern for self, other people, and the biosphere. Journal of Environmental Psychology. 2001;21(4):327–39.

[38] Siegrist M, Árvai J. Risk Perception: Reflections on 40 Years of Research. Risk Analysis. 2020;40(S1):2191–206. doi: 10.1111/risa.13599 32949022

[39] Aldanondo-Ochoa AM, Almansa-Sáez C. The private provision of public environment: Consumer preferences for organic production systems. Land Use Policy. 2009;26(3):669–82.

[40] Palm A. Early adopters and their motives: Differences between earlier and later adopters of residential solar photovoltaics. Renewable and Sustainable Energy Reviews. 2020;133:110142–.

[41] Bredahl L. Determinants of consumer attitudes and purchase intentions with regard to genetically modified foods—results of a cross-national survey. Journal of Consumer Policy. 2001;24(1):23–61.

[42] Vandermoere F, Blanchemanche S, Bieberstein A, Marette S, Roosen J. The public understanding of nanotechnology in the food domain. Public Understanding of Science. 2011;20(2):195–206. 21657134 10.1177/0963662509350139

[43] Borrello M, Cembalo L, Vecchio R. Role of information in consumers’ preferences for eco-sustainable genetic improvements in plant breeding. PLOS ONE. 2021;16(7):e0255130–e. doi: 10.1371/journal.pone.0255130 34324542 PMC8321114

[44] Perito MA, Di Fonzo A, Sansone M, Russo C. Consumer acceptance of food obtained from olive by-products: A survey of Italian consumers. British Food Journal. 2020;122(1):212–26.

[45] Federal Statistical Office. GENESIS-Online. 2020.

[46] Lindblom J, Lundström C, Ljung M, Jonsson A. Promoting sustainable intensification in precision agriculture: review of decision support systems development and strategies. Precision Agriculture. 2017;18(3):309–31.

[47] Bechar A, Vigneault C. Agricultural robots for field operations: Concepts and components. Biosystems Engineering. 2016;149:94–111.

[48] Munnaf MA, Haesaert G, Van Meirvenne M, Mouazen A. Site-specific seeding using multi-sensor and data fusion techniques: A review. Advances in agronomy. 2020;161:241–323.

[49] Lottes P, Behley J, Chebrolu N, Milioto A, Stachniss C. Robust joint stem detection and crop-weed classification using image sequences for plant-specific treatment in precision farming. Journal of Field Robotics. 2020;37(1):20–34.

[50] Federal Ministry of Food Agriculture. Digitalisierung in der Landwirtschaft; Chancen nutzen-Risiken minimieren [Digitization in agriculture; seizing opportunities—minimizing risks]. Bonn 2021.

[51] Rentenbank. Schriftenreihe Der Rentenbank—Band 27 die Gemeinsame Agrarpolitik (GAP) der Europäischen Union nach 2013 [Rentenbank Publication Series—Volume 27 The Common Agricultural Policy (CAP) of the European Union after 2013]. 2013.

[52] Federal Ministry of Food Agriculture. Gemeinsame Agrarpolitik (GAP)—Geschichte der Gemeinsamen Agrarpolitik [Common agricultural policy (CAP)—History of the common agricultural policy].

[53] Siegrist M, Cousin ME, Kastenholz H, Wiek A. Public acceptance of nanotechnology foods and food packaging: The influence of affect and trust. Appetite. 2007;49(2):459–66. doi: 10.1016/j.appet.2007.03.002 17442455

[54] Araki M, Ishii T. Towards social acceptance of plant breeding by genome editing. Trends in plant science. 2015;20(3):145–9. doi: 10.1016/j.tplants.2015.01.010 25726138

[55] Bitkom. Digitalisierung erhöht Akzeptanz für moderne Landwirtschaft [Digitalization increase acceptance of modern agriculture]. 2017.

[56] German Agricultural Society. Digitale Landwirtschaft—Ein Positionspapier der DLG [Digital agriculture—A DLG opinion paper]. 2018.

[57] Yazdanpanah M., and Forouzani M. Application of the Theory of Planned Behaviour to predict Iranian students’ intention to purchase organic food. Journal of Cleaner Production. 2015;107, 342–352.

[58] Inbar Y, Phelps J, Rozin P. Recency negativity: Newer food crops are evaluated less favorably. Appetite. 2020;154:104754–. doi: 10.1016/j.appet.2020.104754 32522592

[59] Rozin P, Spranca M, Krieger Z, Neuhaus R, Surillo D, Swerdlin A, et al. Preference for natural: instrumental and ideational/moral motivations, and the contrast between foods and medicines. Appetite. 2004;43(2):147–54. doi: 10.1016/j.appet.2004.03.005 15458801

[60] Anders S, Cowling W, Pareek A, Gupta KJ, Singla-Pareek SL, Foyer CH. Gaining Acceptance of Novel Plant Breeding Technologies. Trends in Plant Science. 2021;26(6):575–87. doi: 10.1016/j.tplants.2021.03.004 33893048

[61] Mackenzie Podsakoff, Podsakoff. Construct Measurement and Validation Procedures in MIS and Behavioral Research: Integrating New and Existing Techniques. MIS Quarterly. 2011;35(2):293.

[62] Regan Á. Exploring the readiness of publicly funded researchers to practice responsible research and innovation in digital agriculture. Journal of Responsible Innovation. 2021;8(1):28–47.

[63] Rose DC, Chilvers J. Agriculture 4.0: Broadening Responsible Innovation in an Era of Smart Farming. Frontiers in Sustainable Food Systems. 2018;0:87.

